# Association Between Systemic Immune‐Inflammation Index and Psoriasis, Psoriasis Comorbidities, and All‐Cause Mortality: A Study Based on NHANES

**DOI:** 10.1002/iid3.70050

**Published:** 2024-10-28

**Authors:** Yang Zhao, Yan Ping Bai, Lin Feng Li

**Affiliations:** ^1^ Department of Dermatology, Beijing Friendship Hospital Capital Medical University Beijing China; ^2^ Department of Dermatology China‐Japan Friendship Hospital Beijing China

**Keywords:** all‐cause mortality, metabolic syndrome, NHANES, psoriasis, systemic immune‐inflammation index

## Abstract

**Objective:**

The relationship between systemic immune‐inflammation index (SII) and psoriasis and its prognosis is not yet clear. In this study, the correlation between SII and psoriasis, psoriasis comorbidities, and all‐cause mortality was investigated based on the National Health and Nutrition Examination Survey (NHANES).

**Methods:**

The study population was derived from five NHANES cycles: 2003–2006, 2009–2014, and survival follow‐up was as of December 31, 2019. The association between SII and psoriasis and its comorbidities was analyzed using weighted multivariate logistic regression models. Weighted COX regression was used to calculate hazard ratios (HRs) and the corresponding 95% confidence intervals (CIs). Restricted cubic spline, subgroup and sensitivity analyses were also used. Logarithmic conversion was performed on SII(log2SII) to reduce the impact of outliers.

**Results:**

A total of 21,431 participants were included in this study. As a continuous variable, log2SII was significantly associated with psoriasis in the fully adjusted model [OR = 1.20(1.04–1.39), *p* = .01]. log2SII remained positively associated with psoriasis after excluding participants with a history of cancer or cardiovascular disease (CVD), or non‐Hispanic black participants. Among psoriasis patients, log2SII was significantly associated with metabolic syndrome (MetS) [OR = 1.68(1.19,2.38), *p* = .004] and all‐cause mortality [HR = 1.48(1.09,1.99), *p* = .01]. Similar results were consistently observed when SII was analyzed as a categorical variable (in quartiles).

**Conclusion:**

This study suggested a positive association between SII and the prevalence of psoriasis. Among psoriasis patients, SII was positively correlated with MetS and all‐cause mortality.

## Graphical abstract

This study aims to explore the association between SII and Psoriasis, Psoriasis Comorbidities and All‐cause Mortality based on the National Health and Nutrition Examination Survey (NHANES) database. We found that SII was significantly associated with the prevalence of psoriasis. Among psoriasis patients, SII was positively correlated with MetS and all‐cause mortality.

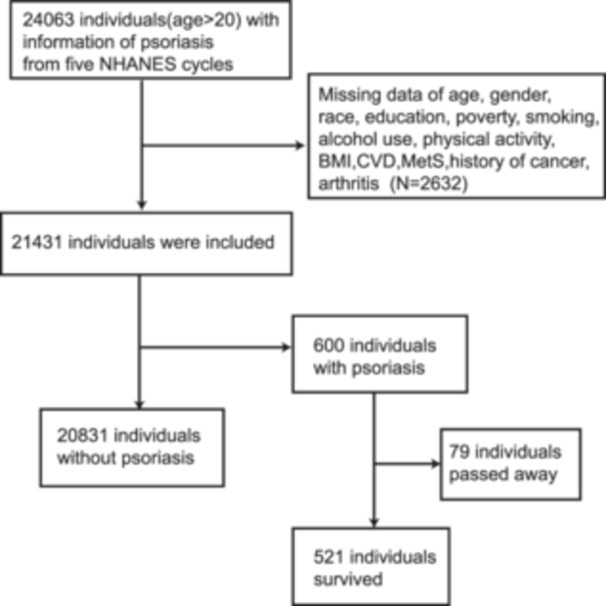

## 1 Introduction

Psoriasis belongs to a systemic inflammatory disease with skin lesions as the main clinical manifestation, and its etiology and pathogenesis are still largely unknown [1, 2]. Compared with the normal population, psoriasis patients are more prone to systemic comorbidities such as CVD, psychological disorders, Crohn's disease, metabolic disorders, etc. due to the presence of persistent inflammation [3]. Studies have found that important inflammatory factors involved in the development and progression of psoriasis (including interleukin 17(IL‐17) and tumor necrosis factor α (TNF‐α), et al.), which are closely related to neutrophils and platelets in innate immunity [4, 5, 6]. Lymphocytes play a crucial role in cytokine production and abnormally elevated neutrophils in psoriasis can secrete a variety of proinflammatory factors (e.g., IL‐17/IL‐6/TNF) to participate in the movement and progression of psoriasis, which in turn can act on neutrophils to promote their chemotaxis, aggregate in the epidermis and dermis, and aggravate the inflammatory damage of psoriasis [7, 8]. Platelets play a central role in immune disorders and inflammatory responses. The important inflammatory factors of psoriasis (IL‐9, IL‐17) are able to activate platelets, leading to their elevated number, which in turn release more thromboxane and increase blood viscosity, resulting in microcirculation disturbance, while secreting inflammatory mediators, promoting neutrophil aggregation, activation, and thus amplifying the inflammatory response [9]. A trend has emerged in medical exercise to make use of inflammation‐based totally indicators (e.g., NLR, MLR, and PLR) as an evaluation technique of disease activity in multiple inflammatory diseases and a prognostic indicator of survival in sufferers with malignancies in current years [10, 11, 12]. SII is a novel inflammatory biomarker composed of neutrophil, lymphocyte, and platelet counts. Given the economy and easy availability of SII, more attention has been paid in predicting the prognosis of infections, tumors, and so on [13, 14, 15, 16, 17, 18, 19, 20, 21, 22]. Although there were some literature reports on the correlation between SII and psoriasis, or the correlation between SII and the efficacy of biological agents in the treatment of psoriasis, the sample sizes of the covered research had been all very small [23, 24]. Therefore, we aimed to examine this association between SII and psoriasis, psoriasis comorbidities, and all‐cause mortality using data from NHANES.

## 2 Methods

### 2.1 Data Sources

NHANES is a countrywide survey that gathers information on the health and dietary conditions of both adults and children across the United States. This survey, conducted by the National Center for Health Statistics (NCHS), utilizes a nationally representative sampling method known as a stratified, multistage likelihood cluster sampling design and undergoes biennial updates. The NHANES 2003–2006 and 2009–2014 cycles provided data on psoriasis, but information on the severity of psoriasis was only available in the 2003–2006 and 2011–2014 cycles. The latest follow‐up date for survival was December 31, 2019, from the National Death Index. The NHANES study protocol was approved by the NCHS research ethics review board, and participants provided written informed consent at enrollment. This study was performed in accordance with the Declaration of Helsinki.

### 2.2 Study Design and Population

**Figure 1:**
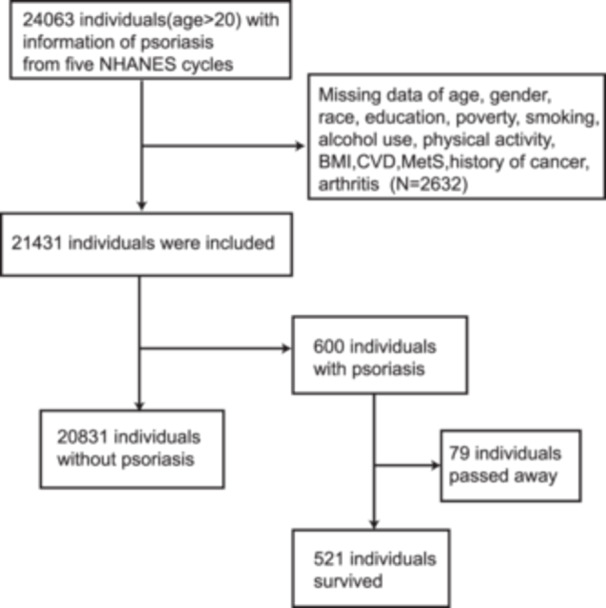
Flowchart of screening study participants from NHANES database.

All participants (24,063, age ≥ 20) were derived from five 2‐year NHANES cycles data (2003–2004, 2005–2006, 2009–2010, 2011–2012, 2013–2014). Exclusion criteria were missing data on age, gender, race, education, poverty, smoking, alcohol use, physical activity, BMI, CVD, MetS, history of cancer, and arthritis (*N* = 2632). A total of 21,431 individuals (20,831 individuals without psoriasis, 600 individuals with psoriasis) with complete information were finally included. As of December 2019, follow‐up information for psoriasis patients showed 521 survivors and 79 deaths. A flow chart of the included population is presented in Figure 1.

### 2.3 Diagnosis of Psoriasis and Calculation Method of SII

The diagnosis of psoriasis relies on questionnaires in the dermatology section or in the medical conditions section: “Have you ever been told by a doctor or other health care professional that you had psoriasis?“ “Yes” stands for patients with psoriasis. The diagnosis of arthropathic psoriasis was made as psoriasis with a history of arthritis. Psoriasis severity was assessed according to the following questionnaires: “Is Psoriasis little or extensive? or Degree of Psoriasis.” The severity of psoriasis was evaluated according to the body surface area (BSA) scoring method [25]. Because the sample size was too small, we stratified psoriasis severity into two grades: (1) Mild psoriasis included “little or no psoriasis” and “only a few patches (that could be covered by one or two palms of your hand)” on the questionnaire. (2) Moderate to severe psoriasis was described as “scattered patches (that could be covered between 3 and 10 palms of your hand) and extensive psoriasis (covering large areas of the body that would be more than ten palms of your hand).” The formula for calculating SII was described as: SII = *N* x *P*/*L* (*N* represents the neutrophil counts, *P* represents the platelet counts, and *L* represents the lymphocyte counts).

### 2.4 Covariates

Based on the literature, the following covariates were included: age, gender, race, education, poverty income ratio, smoking status, drinking status, body mass index (BMI), type of physical activity, metabolic syndrome, and self‐reported history of cancer, CVD, or arthritis. Ethnicity information in the NHANES database was categorized into five groups: Mexican American, Other Hispanic, non‐Hispanic White, non‐Hispanic Black, and other races. Education was divided into three levels: Below high school, High school, and above High school. Smoking status was categorized into three groups: Never (smoked less than 100 cigarettes in life), Former (smoked more than 100 cigarettes in life and no longer smoke), and Now (smoked more than 100 cigarettes in life and smoke some days or every day). Drinking status included the following categories [26]: Never (had < 12 drinks in lifetime), Mild (less than two drinks for males per day and one for females), Moderate (more than three drinks for males per day and two for females, or binge drinking ≥ two & binge < 5 days per month), and Heavy (more than four drinks for males per day and three for females, or binge drinking > 5 days per month). Physical activity levels were classified into three groups: “Inactive” for individuals with no leisure‐time physical activity, “Insufficiently active” for those engaging in moderate activity 1–5 times per week with a metabolic equivalent of task (MET) between 3 and 6, or vigorous activity 1–3 times per week with a MET above 6, and “Active” for individuals reporting a higher frequency of moderate or vigorous activity compared to the other groups mentioned [27]. CVD was defined as coronary heart disease, congestive heart failure, heart attack, stroke, and angina. If the participant had a history of this disease above, they were defined as a CVD case. The participants were asked, “Has a doctor or other health expert ever informed you that you have congestive heart failure/coronary heart disease/angina pectoris/heart attack/stroke?” A person was regarded as having CVD if he or she replied “yes” to any of the above questions [28]. The International Diabetes Federation (IDF) 2009 criteria for metabolic syndrome were employed [29]. History of cancer or arthritis was based on questionnaires: “Doctor ever said you had arthritis” and “Ever told you had cancer or malignancy.”

### 2.5 Statistical Analysis

NHANES analytic guidelines were followed in our analyses, which accounted for the complex sampling design and sampling weights. The sampling weight was computed by dividing the MEC exam weight (wtmec2yr) by five. For continuous variables that exhibited a normal distribution, we reported the results as means along with their corresponding standard errors (SEs). Conversely, for continuous variables that deviated from a normal distribution, we presented the findings as medians accompanied by the interquartile range (IQR). The categorical variables were expressed in frequency (percentage). To compare continuous data, Student's *t*‐test was employed for comparing normally distributed continuous variables, while the Wilcoxon rank‐sum test was employed for non‐normal continuous variables, and the chi‐squared test with Rao & Scott's second‐order correction was utilized for categorical data. The connections between SII and psoriasis, psoriasis comorbidities were examined through the utilization of weighted multivariate logistic regression models. To make the data more in line with a normal distribution and reduce the impact of outliers on the data, we performed a log2 transformation on SII (log2SII). The variable log2SII was evaluated in both its continuous and categorical form in each model. The quartiles of the log2SII were utilized to segregate it into four subgroups, namely Q1, Q2, Q3, and Q4, with Q1 serving as the benchmark group. To account for potential confounding factors, a series of three models were employed for this investigation: Crude model adjusted for nothing, Model I adjusted for age, gender, and ethnicity; Model II adjusted for age gender, ethnicity, education, poverty, body mass index, smoke status, alcohol drinking status, physical activity, and self‐reported history of CVD. Restricted cubic spline plots were used to evaluate the nonlinear trend between SII and psoriasis, psoriasis comorbidities, and all‐cause mortality. Subgroup analyses were performed to evaluate the correlation between SII and psoriasis based on factors such as age, gender, ethnicity, education, poverty, body mass index, smoke status, alcohol drinking status, physical activity, and self‐reported history of CVD. Sensitivity analyses were conducted to evaluate the robustness of our findings, including the exclusion of individuals who were non‐Hispanic black or had a prior history of cancer, CVD or arthritis. Weighted Cox regression was used to explore the correlation between SII and all‐cause mortality in psoriasis patients, and to calculate hazard ratios (HRs), and the corresponding 95% confidence intervals (CIs). Model I adjusted for age, gender; Model II adjusted for age gender, ethnicity, and body mass index; Model III adjusted for age gender, ethnicity, education, poverty, body mass index, smoke status, alcohol drinking status, and self‐reported history of CVD and MetS. Statistical analyses were conducted using the survey package in R version 4.2. A significance level of *p* < 0.05 (two‐sided) was used in all tests to determine statistical significance.

## 3 Results

### 3.1 Characteristics of the Participants

Among the entire study population, 600 participants had psoriasis (3%) and 20,831 participants did not have psoriasis (97%). Based on weight analysis of complex sampling, there were a total of 10,610 males, accounting for 49.12%. The mean age of all participants was 45.02 ± 0.27 years, the mean PIR was 2.99 ± 0.04, the mean BMI was 28.74 ± 0.09 kg/m^2^, the median SII was 481.37(IQR 350.30–671.94). In smoking status, Never, Former, and Now account for 54.35%, 22.88%, and 22.77% respectively. In drinking status, Nondrinker, Low‐to‐moderate drinker, and Heavy drinker account for 15.84%, 74.16%, and 10%, respectively. History of CVD, MetS, arthritis, or cancer account for 7.11%, 32.39%, 22.41%, and 8.41% of the total population, respectively. The above information is presented in Table 1.

**Table 1:** Baseline characteristics of participants with information associated with psoriasis in NHANES 2003–2006 and 2009–2014.

Characteristic	Total (*N* = 21431)	Psoriasis		*p*‐value
		No (N = 20,831)	Yes (N = 600)	
SII	481.37 (350.30,671.94)	478.83 (349.50,668.91)	545.24 (374.00,746.67)	**< 0.001**
Age	45.02 (0.27)	44.94 (0.27)	47.64 (0.75)	**< 0.001**
Poverty income ratio	2.99 (0.04)	2.98 (0.04)	3.14 (0.08)	**0.03**
BMI	28.74 (0.09)	28.70 (0.09)	29.98 (0.33)	**< 0.001**
Neutrophils	4.00 (3.10,5.20)	4.00 (3.10,5.10)	4.30 (3.20,5.50)	**0.01**
Lymphocyte	2.00 (1.60,2.50)	2.00 (1.60,2.50)	2.00 (1.50,2.50)	**0.02**
Platelet	243.00 (206.00,287.00)	243.00 (206.00,287.00)	241.00 (209.00,294.00)	0.51
Sex, %				0.64
Female	10821 (50.88)	10506 (50.85)	315 (51.86)	
Male	10610 (49.12)	10325 (49.15)	285 (48.14)	
Race/ethnicity, %				**< 0.001**
Mexican American	3358 (8.59)	3310 (8.75)	48 (3.70)	
Other Hispanic	1772 (5.16)	1726 (5.20)	46 (3.84)	
Non‐Hispanic White	9553 (68.20)	9183 (67.79)	370 (81.07)	
Non‐Hispanic Black	4589 (11.15)	4514 (11.32)	75 (5.81)	
Other race	2159 (6.90)	2098 (6.95)	61 (5.58)	
Education, %				0.19
Below high school	5200 (16.40)	5080 (16.48)	120 (13.94)	
High school	4869 (22.69)	4735 (22.74)	134 (21.13)	
Above high school	11362 (60.91)	11016 (60.78)	346 (64.93)	
Drinking status, %				0.92
Nondrinker	4175 (15.84)	4064 (15.84)	111 (15.71)	
Low‐to‐moderate drinker	15402 (74.16)	14970 (74.18)	432 (73.72)	
Heavy drinker	1854 (10.00)	1797 (9.98)	57 (10.57)	
Smoking status, %				**< 0.001**
Never smoker	11761 (54.35)	11497 (54.70)	264 (43.32)	
Former smoker	4772 (22.88)	4576 (22.47)	196 (35.48)	
Current smoker	4898 (22.77)	4758 (22.83)	140 (21.19)	
History of CVD, %				**0.01**
No	19521 (92.89)	19009 (92.98)	512 (89.91)	
Yes	1910 (7.11)	1822 (7.02)	88 (10.09)	
History of MetS, %				**0.004**
No	13990 (67.61)	13654 (67.84)	336 (60.46)	
Yes	7441 (32.39)	7177 (32.16)	264 (39.54)	
Physical activity, %				0.83
Inactive	5376 (21.04)	5221 (21.03)	155 (21.24)	
Insufficiently active	8032 (40.98)	7801 (40.94)	231 (42.40)	
Active	8023 (37.98)	7809 (38.03)	214 (36.36)	
History of arthritis %				**< 0.001**
No	16365 (77.59)	16012 (78.08)	353 (62.03)	
Yes	5066 (22.41)	4819 (21.92)	247 (37.97)	
History of cancer %				**< 0.001**
No	19740 (91.59)	19219 (91.74)	521 (86.90)	
Yes	1691 (8.41)	1612 (8.26)	79 (13.10)	

*Note:* Normally distributed continuous variables are described as means ± SEs, and continuous variables without a normal distribution are presented as medians [interquartile ranges]. Categorical variables are presented as numbers (percentages). *N* reflect the study sample. The use of bold font is to show that the *p*‐value of the statistic is less than 0.05, making it more prominent.

Abbreviations: BMI, Body mass index (kg/m^2^); CVD, cardiovascular disease; MetS, metabolic syndrome; SII, systemic immune‐inflammation index.

### 3.2 Association Between SII and Psoriasis

**Figure 2:**
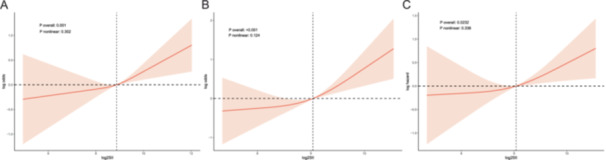
Analysis of restricted cubic spline regression of the relationship between log2SII and the prevalence of psoriasis, MetS and all‐cause mortality. (A) RCS plot of log2SII and the prevalence of psoriasis; (B) RCS plot of log2SII and MetS; (C) RCS plot of log2SII and all‐cause mortality), Adjusted for age gender, ethnicity, plus marital status, education, poverty, body mass index, smoke status, alcohol drinking status, physical activity and self‐reported history of CVD. SII, systemic immune‐inflammation index; CV, cardiovascular disease.

**Figure 3:**
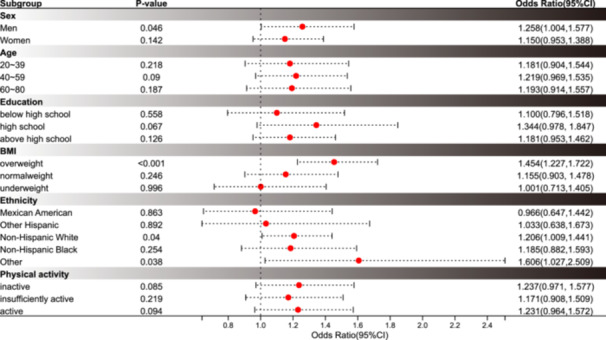
Subgroup analysis of the association between SII and the incidence of psoriasis. All stratifications were adjusted for age, gender, ethnicity, education, poverty, body mass index, smoking status, alcohol drinking status, physical activity and self‐reported history of CVD, except for the stratification factor itself. SII, systemic immune‐inflammation index; CVD, cardiovascular disease.

The multivariate regression analyses with sample weights are summarized in Table 2. Association between SII and Psoriasis was shown in Model II (the fully adjusted model) [OR = 1.20(1.04–1.39), *p* = 0.01]. When SII was divided into quartiles, with the lowest quartile Q1 as the reference, SII was significantly associated with psoriasis (Q 4 vs Q1: OR, 1.34; 95% CI, 0.96–1.88; *P* for trend = 0.007). Multivariate adjusted RCS confirmed that there was a linear relationship between SII and psoriasis (Figure 2A). Subgroup analysis of the association between SII and psoriasis is exhibited in Figure 3. Among male patients [OR = 1.26(1.00–1.58) *p* = 0.046], non‐Hispanic white [OR = 1.21(1.01–1.44), *p* = 0.04], Other race [OR = 1.61(1.03‐2.51), *p* = .038] and Obesity [OR = 1.45(1.23‐1.72), *p* < 0.001], SII was significantly associated with psoriasis when adjusted for age, gender, ethnicity, education, poverty, body mass index, smoking status, alcohol drinking status, physical activity and self‐reported history of CVD apart from stratification factor itself.

**Table 2:** Association between systemic immune‐inflammation index (SII) and psoriasis.

SII	Crude model		Model I		Model II	
	OR (95%CI)	*p*	OR (95%CI)	*p*	OR (95%CI)	*p*
Continuous variable (log2SII)	1.30 (1.13,1.49)	**< 0.001**	**1.24 (1.08,1.43)**	**0.003**	**1.20 (1.04,1.39)**	**0.01**
Categorical variable						
Q1	Reference		Reference		Reference	
Q2	0.84 (0.57,1.24)	0.39	0.80 (0.54,1.19)	0.26	0.79 (0.53,1.18)	0.24
Q3	1.29 (0.92,1.80)	0.13	1.21 (0.86,1.69)	0.28	1.17 (0.83,1.66)	0.35
Q4	**1.55 (1.11,2.17)**	**0.01**	**1.42 (1.01,2.00)**	**0.04**	1.34 (0.96,1.88)	0.09
P for trend		**< 0.001**		**0.002**		**0.007**

*Note:* Crude model adjusted for nothing, Model I adjusted for age, gender, and ethnicity; Model II adjusted for age, gender, ethnicity, education, poverty, body mass index, smoke status, alcohol drinking status, physical activity, and self‐reported history of CVD. Q1 Range, [1.53, 333.14]; Q2 Range, (333.14,467.79]; Q3 Range, (467.79,656.10]; Q4 Range (656.1,28397.28]. The use of bold font is to show that the *p*‐value of the statistic is less than 0.05, making it more prominent.

### 3.3 Sensitivity Analysis

SII remained significantly associated with psoriasis after excluding participants with a history of cancer [OR = 1.24 (1.06–1.45), *p* = 0.01]. Excluding participants with a history of CVD, SII remained significantly associated with psoriasis [OR = 1.26 (1.08–1.47), *p* = 0.004]. Excluding non‐Hispanic black participants, SII was also significantly associated with psoriasis [OR = 1.20 (1.03–1.40), *p* = 0.02]. When SII was divided into quartiles, the trend remained significant (*P* for trend< 0.05). The results are summarized in Table 3.

**Table 3:** Sensitivity analyses.

Analysis	Crude Model		Model I		Model II	
	OR (95%CI)	*p*	OR (95%CI)	*p*	OR (95%CI)	*p*
Excluding participants with a history of cancer						
Continuous variable (log2SII)	**1.33 (1.15,1.55)**	**< 0.001**	**1.28 (1.10,1.49)**	**0.002**	**1.24 (1.06,1.45)**	**0.01**
Categorical variable						
Q1 Range [1.529,331.734]	Reference		Reference		Reference	
Q2 Range (331.734,464.578]	0.84 (0.57,1.24)	0.37	0.79 (0.53,1.18)	0.24	0.78 (0.52,1.16)	0.21
Q3 Range (464.578,651.523]	1.31 (0.93,1.86)	0.12	1.22 (0.86,1.74)	0.26	1.20 (0.84,1.71)	0.32
Q4 Range (651.523,28397.276]	**1.56 (1.12,2.18)**	**0.01**	**1.43 (1.02,2.00)**	**0.04**	1.34 (0.96,1.88)	0.08
P for trend		**< 0.001**		**0.002**		**0.007**
Excluding participants with a history of CVD						
Continuous variable (log2SII)	1.35 (1.15,1.58)	**< 0.001**	**1.30 (1.11,1.52)**	**0.002**	**1.26 (1.08,1.47)**	**0.004**
Categorical variable						
Q1 Range [1.529,333]	Reference		Reference		Reference	
Q2 Range (333,464.933]	0.90 (0.58,1.39)	0.62	0.84 (0.54,1.32)	0.44	0.83 (0.53,1.31)	0.42
Q3 Range (464.933,650.903]	1.40 (0.96,2.04)	0.08	1.29 (0.88,1.90)	0.18	1.26 (0.86,1.86)	0.23
Q4 Range (650.903,28397.276]	1.67 (1.12,2.48)	0.01	1.52 (1.02,2.27)	0.04	1.45 (0.98,2.15)	0.06
P for trend		**< 0.001**		**0.002**		**0.005**
Excluding non‐Hispanic black participants						
Continuous variable (log2SII)	**1.26 (1.08,1.48)**	**0.003**	**1.24 (1.07,1.45)**	**0.01**	**1.20 (1.03,1.40)**	**0.02**
Categorical variable						
Q1 Range [1.529,353.223]	Reference		Reference		Reference	
Q2 Range (353.223,486.91]	0.75 (0.50,1.11)	0.15	0.74 (0.49,1.10)	0.13	0.73 (0.49,1.09)	0.12
Q3 Range (486.91,675]	**1.44 (1.01,2.03)**	**0.04**	1.42 (1.01,2.02)	0.05	1.37 (0.96,1.95)	0.08
Q4 Range (675,28397.276]	**1.50 (1.06,2.12)**	**0.02**	**1.45 (1.03,2.06)**	**0.04**	1.37 (0.97,1.95)	0.08
P for trend		**< 0.001**		**< 0.001**		**0.003**

*Note:* Crude model adjusted for nothing, Model I adjusted for age, gender, and ethnicity; Model II adjusted for age, gender, ethnicity, education, poverty, body mass index, smoke status, alcohol drinking status, physical activity, self‐reported history of CVD, except for the stratification factor itself. The use of bold font is to show that the *p*‐value of the statistic is less than 0.05, making it more prominent.

### 3.4 Association Between SII and Psoriasis Severity

A total of 429 participants with psoriasis severity information were collected in the 2003–2006 and 2011–2014 cycles. There were 349 participants with mild psoriasis and 80 participants with moderate to severe psoriasis (baseline characteristics are summarized in Table 1). Multivariate regression analysis showed that SII was not significantly associated with BSA severity of psoriasis whether as a continuous variable or a categorical variable (*p* > 0.05) (Table 2).

### 3.5 Association Between SII and Psoriasis Comorbidities

Among 600 psoriasis patients, 336 did not have metabolic syndrome, and 224 had metabolic syndrome (baseline characteristics are presented in Table 3). The incidence of metabolic syndrome was as high as 44%. A significant difference could be observed in SII levels (*p* < 0.001). Both as continuous variables [OR = 1.68(1.19–2.38), *p* = .004] and as categorical variables (Q4 vs Q1: OR, 2.49; 95% CI, 1.17–5.30; *P* for trend = 0.041), SII was significantly correlated with MetS among psoriasis patients (The above information is in Table 4). Multivariate restricted cubic spline regression showed a linear relationship between SII and MetS in psoriasis patients when adjusted for age, gender, ethnicity, education, poverty, body mass index, smoking status, alcohol drinking status, physical activity, and self‐reported history of CVD (Figure 2B). Although the fully adjusted RCS showed a linear relationship between SII and arthritis (Figure 1) and CVD (Figure 2), multivariate regression analysis showed that SII was not significantly associated with arthropathic psoriasis or CVD (*p* > 0.05) (Table 4).

**Table 4:** Correlation analysis between SII and MetS among psoriasis patients.

Analysis	Crude model		Model I		Model II	
	OR (95%CI)	*p*	OR (95%CI)	*p*	OR (95%CI)	*p*
Correlation analysis between SII and MetS						
Continuous variable (log2SII)	1.75 (1.36,2.27)	**< 0.001**	**1.86 (1.39,2.48)**	**< 0.001**	**1.68 (1.19,2.38)**	**0.004**
Categorical variable						
Q1	Reference		Reference		Reference	
Q2	1.26 (0.74,2.17)	0.39	1.23 (0.66,2.29)	0.51	1.23 (0.63,2.38)	0.54
Q3	1.24 (0.70,2.18)	0.46	1.36 (0.71,2.62)	0.35	1.18 (0.59,2.37)	0.64
Q4	**2.99 (1.72,5.22)**	**< 0.001**	**3.36 (1.75,6.46)**	**< 0.001**	**2.49 (1.17,5.30)**	**0.02**
P for trend		**< 0.001**		**0.001**		**0.041**

*Note:* Crude model adjusted for nothing, Model I adjusted for age, gender, and ethnicity; Model II adjusted for age, gender, ethnicity, education, poverty, body mass index, smoking status, alcohol drinking status, physical activity and self‐reported history of CVD. Q1 Range, [92,366.667]; Q2 Range, (366.667,525.709]; Q3 Range, (525.709,746.069]; Q4 Range, (746.069,3131]. The use of bold font is to show that the *p*‐value of the statistic is less than 0.05, making it more prominent.

### 3.6 Association Between SII and All‐Cause Mortality Among Psoriasis Patients

Three models were used to investigate the correlation between SII and all‐cause mortality in psoriasis patients (Table 5). Model I adjusted for age, gender; Model II adjusted for age, gender, ethnicity, and body mass index; Model III adjusted for age, gender, ethnicity, education, poverty, body mass index, smoking status, alcohol drinking status and self‐reported history of CVD, and MetS. In fully adjusted models, SII was significantly correlated with all‐cause mortality in psoriasis patients [HR = 1.48(1.09, 1.99), *p* = 0.01]. SII, as a categorical variable, yielded consistent results (Q 4 vs Q1: OR, 1.92; 95% CI, 1.02–3.60; *p* for trend = 0.007). Multivariate restricted cubic spline regression showed a linear relationship between SII and all‐cause mortality among psoriasis patients (Figure 2C).

**Table 5:** Correlation analysis between SII and all‐cause mortality among psoriasis patients.

Analysis	Model I		Model II		Model III	
	OR (95%CI)	*p*	OR (95%CI)	*p*	OR (95%CI)	*p*
Correlation analysis between SII and mortality						
Continuous variable (log2SII)	1.63 (1.13,2.36)	**0.01**	**1.62 (1.16, 2.25)**	**0.004**	**1.48 (1.09, 1.99)**	**0.01**
Categorical variable						
Q1	Reference		Reference		Reference	
Q2	1.25 (0.57,2.73)	0.58	1.27 (0.58, 2.78)	0.55	1.23 (0.53, 2.88)	0.63
Q3	1.02 (0.47,2.23)	0.96	0.99 (0.45, 2.17)	0.98	1.00 (0.44, 2.29)	1.00
Q4	**2.26 (1.09,4.68)**	**0.03**	**2.17 (1.09, 4.31)**	**0.03**	**1.92 (1.02, 3.60)**	**0.04**
*p* for trend		**0.048**		**0.048**		**0.007**

*Note:* Model I adjusted for age, gender; Model II adjusted for age, gender, ethnicity, body mass index; Model III adjusted for age, gender, ethnicity, education, poverty, body mass index, smoking status, alcohol drinking status and self‐reported history of CVD and MetS. Q1 Range, [92,366.667]; Q2 Range, (366.667,525.709]; Q3 Range, (525.709,746.069]; Q4 Range, (746.069,3131]. The use of bold font is to show that the *p*‐value of the statistic is less than 0.05, making it more prominent.

## 4 Discussion

Psoriasis is a persistent recurring skin condition that impacts around 7.5 million adults in the United States, and the prevalence is increasing year by year, becoming a large socioeconomic burden [30, 31]. Inflammation in psoriasis already extends beyond the skin and affects joints, cardiovascular and neurological systems [32]. Despite psoriasis being a systemic inflammatory skin disease, conventional inflammatory markers such as CRP, ESR, etc., do not exhibit elevated levels [33, 34]. Currently, there is no optimal inflammatory marker for psoriasis and its prognosis. Recent research has revealed a novel indicator for inflammation and immunity i.e. low‐cost and accessible: the systemic immune‐inflammation index (SII index) [16, 18, 35, 36]. As an inflammatory biomarker, SII is significantly associated with hidradenitis suppurativa, acne, urticaria and other diseases in the field of dermatology [37]. For moderate to severe acne, SII can be used as a biomarker of the anti‐inflammatory effect of isotretinoin [38]. SII can serve as a new biomarker for predicting the response of chronic spontaneous urticaria to omazumab [39]. The SII level of Behçet disease during the activity period is relatively high, and SII can be used as an additional indicator to evaluate the status of Behçet disease [40]. It has been suggested that SII, in combination with IgE, may help to identify early responders to dupilumab and to develop alternative treatment options for late or non‐responders [41]. But only a small amount of literature has investigated the association between SII and psoriasis, and most of them were single‐center studies with very small sample size.

In this nationally representative survey, the weighted prevalence of psoriasis was 3%, and positively significant associations between SII and psoriasis were found among the US adults with psoriasis. This was consistent with what has been reported in former literature [24]. Psoriasis is an immune‐mediated systemic inflammatory skin disease, and multiple inflammatory factors are involved in its pathogenesis. SII is calculated through neutrophils, platelets, and lymphocytes, which are involved in the inflammatory mechanism of psoriasis.

Neutrophils and platelets have also been suggested as potential contributors to systemic inflammation in psoriasis [42]. Neutrophils migrate into psoriatic lesions to enhance inflammation by elevating oxidative stress. Increased cytokines at the site of inflammation can directly activate platelets, which in turn promote thrombosis and potentiate the inflammatory response [43]. It also illustrated that platelets and neutrophils were clinically relevant, and thus their combined important role in psoriatic inflammation and immune regulation. Therefore, SII can be used to reflect the level of systematic inflammation in psoriasis patients to some extent. Subgroup analysis showed that the ORs of the association between SII and psoriasis were higher among male, non‐Hispanic white, other race or overweight patients. SII remained positively significantly associated with psoriasis after excluding participants with history of cancer, CVD or non‐Hispanic black participants.

It had been reported in the literature that SII is significantly correlated with the severity and mobility of psoriasis and had a high diagnostic value for the severity of psoriasis [23, 44]. But the results of this study were contrary, after multivariate regression analysis incorporating sampling weights, it was found that there was no significant association between SII and the BSA severity of psoriasis. There might be reasons for the inconsistent findings: (1) The study populations were not the same, this study was conducted on adult psoriatic patients in the United States and contained a multiethnic population. (2) Sample size was not the same, this study was conducted based on NHANES data and belonged to a complex sampling stratification study. It represented the health and nutritional status of the whole American population. (3) Psoriasis severity was assessed by different methods, and this study was based on questionnaires, which were evaluated according to BSA evaluation criteria. Previous studies evaluated psoriasis severity or disease activity according to the PASI assessment method.

Numerous studies have shown that psoriasis is associated with many comorbidities, including metabolic syndrome, cardiovascular disease, gastrointestinal disease, arthritis, kidney disease, as well as malignant tumors and psychological disorders [45]. Among the above comorbidities, cardiovascular diseases are particularly important as they often directly affect the mortality rate of patients [46]. This study analyzed the correlation between SII and metabolic syndrome, cardiovascular disease, and psoriatic arthritis in patients with psoriasis. The results confirmed that the incidence of metabolic syndrome significantly increased in the population of psoriasis patients, reaching 44%, consistent with the results reported in the literature [47, 48]. SII was significantly positively correlated with metabolic syndrome, but not with CVD or psoriatic arthritis, although it had been reported that SII was significantly associated with arthropathic psoriasis and could reflect the severity of joint damage [24, 49]. This might suggest that systemic inflammation in psoriasis was not solely determined by the area of skin damage or joint damage, but might also be largely related to psoriasis comorbidities such as MetS. SII was significantly positively associated with the all‐cause mortality rate in psoriasis patients, indicating that inflammation was a critical factor in the all‐cause mortality of psoriasis patients. Proactive anti‐inflammatory treatment might be an important measure to improve the prognosis of psoriasis patients and the emerging biologics for the treatment of psoriasis in recent years, targeting inflammatory pathways, also well demonstrated the importance of anti‐inflammatory therapy for psoriasis [50, 51, 52, 53, 54, 55].

The significantly positive correlation between SII and the incidence, comorbidity, and all‐cause mortality of psoriasis suggested that inflammation ran through the process of psoriasis occurrence and development. The understanding of psoriasis has risen from simple erythematous scaling skin disease to chronic recurrent systemic inflammatory disease. The shared genetic background, concurrent chronic inflammatory processes, and disrupted immune regulatory mechanisms may underlie the development of multiple comorbidities in psoriasis. The attention to comorbidities of psoriasis is increasing. SII, as an easily accessible and inexpensive indicator in clinical practice, has certain clinical value in predicting whether psoriasis patients are accompanied by metabolic syndrome and their survival prognosis.

This study also has certain limitations: (1) In the NHANES database, the diagnosis of psoriasis was mainly self‐reported, which could cause memory bias and lead to a lower incidence of psoriasis than the actual data. (2) Due to the limited number of psoriasis patients and the fact that severity evaluation was mainly based on BSA evaluation criteria, there was a lack of relevant information for PASI evaluation, which may be an important factor that was inconsistent with previous literature reports. (3) There may be other confounding factors (such as respiratory disease, autoimmune diseases, etc.), but they had not been adjusted in this study. However, the advantage of NHANES data is its nationwide representativeness, which makes our research results more reliable.

## 5 Conclusions

SII was positively correlated with psoriasis, and there was a positive correlation could be observed between SII and metabolic syndrome and all‐cause mortality in psoriasis patients. SII might serve as a convenient biomarker for monitoring MetS and all‐cause mortality among patients with psoriasis.

## Author Contributions

**Lin Feng Li:** Conceptualization, Investigation, Methodology. **Yang Zhao:** Data processing, Software, Writing draft manuscript. **Yan Ping Bai:** Visualization, Data curation Supervision.

## Ethics Statement

The NHANES research protocols were approved by the NCHS Research Ethics Review Board, and all participants provided their informed consent in writing.

## Conflicts of Interest

The authors declare no conflicts of interest.

## Supporting information

Supporting information.

## Data Availability

The data generated and/or analyzed in this study is available through the following link: https://wwwn.cdc.gov/nchs/nhanes/default.aspx.

## References

[1] Y. Deng , C. Chang , and Q. Lu , “The Inflammatory Response in Psoriasis: a Comprehensive Review,” Clinical Reviews in Allergy & Immunology 50, no. 3 (2016): 377–389. 27025861 10.1007/s12016-016-8535-x

[2] J. Zeng , S. Luo , Y. Huang , and Q. Lu , “Critical role of environmental factors in the pathogenesis of psoriasis,” The Journal of Dermatology 44, no. 8 (2017): 863–872. 28349593 10.1111/1346-8138.13806

[3] Y. Wang , P. Zhang , Y. Lv , et al., “Advancements in the Study of Biologic Agents in Comorbidities of Psoriasis: A Literature Review,” Clinical, Cosmetic and Investigational Dermatology 16 (2023): 3487–3495. 38077921 10.2147/CCID.S439110 PMC10706046

[4] C. Seignez and M. Phillipson , “The multitasking neutrophils and their involvement in angiogenesis,” Current Opinion in Hematology 24, no. 1 (2017): 3–8. 27755126 10.1097/MOH.0000000000000300

[5] J. L. Harden , J. G. Krueger , and A. M. Bowcock , “The immunogenetics of Psoriasis: A comprehensive review,” Journal of Autoimmunity 64 (2015): 66–73. 26215033 10.1016/j.jaut.2015.07.008 PMC4628849

[6] E. Trovato , P. Rubegni , and E. Cinotti , “The Immunogenetics of Psoriasis,” Adv Exp Med Biol 1367 (2022): 105–117. 35286693 10.1007/978-3-030-92616-8_4

[7] A. Song , S. E. Lee , and J. H. Kim , “Immunopathology and Immunotherapy of Inflammatory Skin Diseases,” Immune Network 22, no. 1 (2022): e7. 35291649 10.4110/in.2022.22.e7 PMC8901701

[8] C. S. B. Andersen , A. Kvist‐Hansen , M. Siewertsen , et al., “Blood Cell Biomarkers of Inflammation and Cytokine Levels as Predictors of Response to Biologics in Patients with Psoriasis,” International Journal of Molecular Sciences 24 (2023): 6111. 37047086 10.3390/ijms24076111 PMC10094459

[9] J. Yuan , P. Ding , M. Yu , et al., “IL‐17 Induces MPTP opening through ERK2 and P53 signaling pathway in human platelets,” Journal of Huazhong University of Science and Technology [Medical Sciences] 35, no. 5 (2015): 679–683. 26489621 10.1007/s11596-015-1489-z

[10] J. Liu , S. Li , S. Zhang , et al., “Systemic immune‐inflammation index, neutrophil‐to‐lymphocyte ratio, platelet‐to‐lymphocyte ratio can predict clinical outcomes in patients with metastatic non‐small‐cell lung cancer treated with nivolumab,” Journal of Clinical Laboratory Analysis 33, no. 8 (2019): e22964. 31282096 10.1002/jcla.22964 PMC6805305

[11] K. Gao , W. Zhu , W. Liu , et al., “Diagnostic value of the blood monocyte‐lymphocyte ratio in knee osteoarthritis,” Journal of International Medical Research 47, no. 9 (2019): 4413–4421. 31342819 10.1177/0300060519860686 PMC6753563

[12] M. Pilaczyńska‐Cemel , R. Gołda , A. Dąbrowska , and G. Przybylski , “Analysis of the level of selected parameters of inflammation, circulating immune complexes, and related indicators (neutrophil/lymphocyte, platelet/lymphocyte, CRP/CIC) in patients with obstructive diseases,” Central European Journal of Immunology 44, no. 3 (2019): 292–298. 31871418 10.5114/ceji.2019.87498 PMC6925570

[13] J. H. Chen , E. T. Zhai , Y. J. Yuan , et al., “Systemic immune‐inflammation index for predicting prognosis of colorectal cancer,” World Journal of Gastroenterology 23, no. 34 (2017): 6261–6272. 28974892 10.3748/wjg.v23.i34.6261 PMC5603492

[14] Y. Ji and H. Wang , “Prognostic prediction of systemic immune‐inflammation index for patients with gynecological and breast cancers: a meta‐analysis,” World Journal of Surgical Oncology 18, no. 1 (2020): 197. 32767977 10.1186/s12957-020-01974-w PMC7414550

[15] M. Li , Z. Li , Z. Wang , C. Yue , W. Hu , and H. Lu , “Prognostic value of systemic immune‐inflammation index in patients with pancreatic cancer: a meta‐analysis,” Clinical and Experimental Medicine 22, no. 4 (2022): 637–646. 35022918 10.1007/s10238-021-00785-x

[16] X. Li , S. Zhang , J. Lu , C. Li , and N. Li , “The prognostic value of systemic immune‐inflammation index in surgical esophageal cancer patients: An updated meta‐analysis,” Frontiers in Surgery 9 (2022): 922595. 36090319 10.3389/fsurg.2022.922595 PMC9459851

[17] L. Meng , Y. Yang , X. Hu , R. Zhang , and X. Li , “Prognostic value of the pretreatment systemic immune‐inflammation index in patients with prostate cancer: a systematic review and meta‐analysis,” Journal of Translational Medicine 21, no. 1 (2023): 79. 36739407 10.1186/s12967-023-03924-y PMC9898902

[18] X. Peng , X. Wang , L. Hua , and R. Yang , “Prognostic and Clinical Value of the Systemic Immune‐Inflammation Index in Biliary Tract Cancer: A Meta‐Analysis,” Journal of Immunology Research 2022 (2022): 1–11. 10.1155/2022/6988489 PMC9691295 36438200

[19] Y. Qiu , Z. Zhang , and Y. Chen , “Prognostic Value of Pretreatment Systemic Immune‐Inflammation Index in Gastric Cancer: A Meta‐Analysis,” Frontiers in Oncology 11 (2021): 537140. 33777726 10.3389/fonc.2021.537140 PMC7990885

[20] Y. Wang , Y. Li , P. Chen , W. Xu , Y. Wu , and G. Che , “Prognostic value of the pretreatment systemic immune‐inflammation index (SII) in patients with non‐small cell lung cancer: a meta‐analysis,” Annals of Translational Medicine 7, no. 18 (2019): 433. 31700869 10.21037/atm.2019.08.116 PMC6803224

[21] R. Yang , Q. Chang , X. Meng , N. Gao , and W. Wang , “Prognostic value of Systemic immune‐inflammation index in cancer: A meta‐analysis,” Journal of Cancer 9, no. 18 (2018): 3295–3302. 30271489 10.7150/jca.25691 PMC6160683

[22] Y. Zhang , Y. Sun , and Q. Zhang , “Prognostic value of the systemic immune‐inflammation index in patients with breast cancer: a meta‐analysis,” Cancer Cell International 20 (2020): 224. 32528232 10.1186/s12935-020-01308-6 PMC7282128

[23] D. Dincer Rota and E. Tanacan , “The utility of systemic‐immune inflammation index for predicting the disease activation in patients with psoriasis,” International Journal of Clinical Practice 75, no. 6 (2021): e14101. 33619821 10.1111/ijcp.14101

[24] A. Yorulmaz , Y. Hayran , U. Akpinar , and B. Yalcin , “Systemic Immune‐Inflammation Index (SII) Predicts Increased Severity in Psoriasis and Psoriatic Arthritis,” Current health sciences journal 46, no. 4 (2020): 352–357. 33717509 10.12865/CHSJ.46.04.05 PMC7948012

[25] B. Strober , C. Ryan , P. van de Kerkhof , et al., “Recategorization of psoriasis severity: Delphi consensus from the International Psoriasis Council,” Journal of the American Academy of Dermatology 82, no. 1 (2020): 117–122. 31425723 10.1016/j.jaad.2019.08.026

[26] P. Rattan , D. D. Penrice , J. C. Ahn , et al., “Inverse Association of Telomere Length With Liver Disease and Mortality in the US Population,” Hepatology Communications 6, no. 2 (2022): 399–410. 34558851 10.1002/hep4.1803 PMC8793996

[27] S. Beddhu , B. C. Baird , J. Zitterkoph , J. Neilson , and T. Greene , “Physical activity and mortality in chronic kidney disease (NHANES III),” Clinical Journal of the American Society of Nephrology 4, no. 12 (2009): 1901–1906. 19820134 10.2215/CJN.01970309 PMC2798872

[28] K. Dang , X. Wang , J. Hu , et al., “The association between triglyceride‐glucose index and its combination with obesity indicators and cardiovascular disease: NHANES 2003‐2018,” Cardiovascular Diabetology 23, no. 1 (2024): 8. 38184598 10.1186/s12933-023-02115-9 PMC10771672

[29] K. G. Alberti , R. H. Eckel , S. M. Grundy , et al., “Harmonizing the metabolic syndrome: a joint interim statement of the International Diabetes Federation Task Force on Epidemiology and Prevention; National Heart, Lung, and Blood Institute; American Heart Association; World Heart Federation; International Atherosclerosis Society; and International Association for the Study of Obesity,” Circulation. 120, no. 16 (2009): 1640–1645. 19805654 10.1161/CIRCULATIONAHA.109.192644

[30] A. W. Armstrong , M. D. Mehta , C. W. Schupp , G. C. Gondo , S. J. Bell , and C. E. M. Griffiths , “Psoriasis Prevalence in Adults in the United States,” JAMA Dermatology 157, no. 8 (2021): 940–946. 34190957 10.1001/jamadermatol.2021.2007 PMC8246333

[31] J. Vanderpuye‐Orgle , Y. Zhao , J. Lu , et al., “Evaluating the economic burden of psoriasis in the United States,” Journal of the American Academy of Dermatology 72, no. 6 (2015): 961–967 e965. 25882886 10.1016/j.jaad.2015.02.1099

[32] D. R. Jadon , C. Stober , S. R. Pennington , and O. FitzGerald , “Applying precision medicine to unmet clinical needs in psoriatic disease,” Nature Reviews Rheumatology 16, no. 11 (2020): 609–627. 33024296 10.1038/s41584-020-00507-9

[33] C. T. Ritchlin , R. A. Colbert , and D. D. Gladman , “Psoriatic Arthritis,” The New England Journal of Medicine 376, no. 21 (2017): 2095–2096. 10.1056/NEJMc1704342 28538114

[34] J. Wu , L. Yan , and K. Chai , “Systemic immune‐inflammation index is associated with disease activity in patients with ankylosing spondylitis,” Journal of Clinical Laboratory Analysis 35, no. 9 (2021): e23964. 34418163 10.1002/jcla.23964 PMC8418483

[35] B. Hu , X. R. Yang , Y. Xu , et al., “Systemic immune‐inflammation index predicts prognosis of patients after curative resection for hepatocellular carcinoma,” Clinical Cancer Research 20, no. 23 (2014): 6212–6222. 25271081 10.1158/1078-0432.CCR-14-0442

[36] Y. Geng , Y. Shao , D. Zhu , et al., “Systemic Immune‐Inflammation Index Predicts Prognosis of Patients with Esophageal Squamous Cell Carcinoma: A Propensity Score‐matched Analysis,” Scientific Reports 6 (2016): 39482. 28000729 10.1038/srep39482 PMC5175190

[37] T. Gambichler , S. Hessam , P. Cramer , N. Abu Rached , and F. G. Bechara , “Complete blood collection‐based systemic inflammation biomarkers for patients with hidradenitis suppurativa,” Journal of the European Academy of Dermatology and Venereology 36, no. 9 (2022): 1593–1596. 35462426 10.1111/jdv.18175

[38] Ç. Turan and N. Metin , “A Novel Inflammatory Marker in the Follow‐up of Moderate‐to‐Severe Acne Vulgaris Administered Isotretinoin: Systemic Immune‐Inflammation Index (SII),” Current health sciences journal 48, no. 1 (2022): 63–67. 35911945 10.12865/CHSJ.48.01.09 PMC9289579

[39] N. C. Cosansu , R. O. Kara , M. Yaldiz , and B. S. Dikicier , “New markers to predict the response to omalizumab in chronic spontaneous urticaria,” Dermatol Ther 35, no. 8 (2022): e15589. 35582853 10.1111/dth.15589

[40] E. Tanacan , D. Dincer , F. G. Erdogan , and A. Gurler , “A cutoff value for the Systemic Immune‐Inflammation Index in determining activity of Behçet disease,” Clinical and Experimental Dermatology 46, no. 2 (2021): 286–291. 32869876 10.1111/ced.14432

[41] A. Zinellu , F. Sucato , V. Piras , et al., “Blood Cells Count Derived Inflammation Indexes as Predictors of Early Treatment Response to Dupilumab in Patients with Moderate‐to‐Severe Atopic Dermatitis,” Journal of Clinical Medicine 12 (2023): 2104. 36983107 10.3390/jcm12062104 PMC10056555

[42] E. Sugimoto , H. Matsuda , S. Shibata , et al., “Impact of Pretreatment Systemic Inflammatory Markers on Treatment Persistence with Biologics and Conventional Systemic Therapy: A Retrospective Study of Patients with Psoriasis Vulgaris and Psoriatic Arthritis,” Journal of Clinical Medicine 12, no. 8 (2023): 3046. 37109382 10.3390/jcm12083046 PMC10145777

[43] T. J. Barrett , M. Schlegel , F. Zhou , et al., “Platelet regulation of myeloid suppressor of cytokine signaling 3 accelerates atherosclerosis,” Science Translational Medicine 11, no. 517 (2019): eaax0481, 10.1126/scitranslmed.aax0481. 31694925 PMC6905432

[44] O. M. Tiucă , S. H. Morariu , C. R. Mariean , R. A. Tiucă , A. C. Nicolescu , and O. S. Cotoi , “Impact of Blood‐Count‐Derived Inflammatory Markers in Psoriatic Disease Progression,” Life (Basel) 14, no. 1 (2024): 114, 10.3390/life14010114. 38255729 PMC10820213

[45] J. Takeshita , S. Grewal , S. M. Langan , et al., “Psoriasis and comorbid diseases,” Journal of the American Academy of Dermatology 76, no. 3 (2017): 377–390. 28212759 10.1016/j.jaad.2016.07.064 PMC5731650

[46] W. H. Boehncke , “Systemic Inflammation and Cardiovascular Comorbidity in Psoriasis Patients: Causes and Consequences,” Frontiers in Immunology 9 (2018): 579. 29675020 10.3389/fimmu.2018.00579 PMC5895645

[47] P. Gisondi , A. C. Fostini , I. Fossà , G. Girolomoni , and G. Targher , “Psoriasis and the metabolic syndrome,” Clinics in Dermatology 36, no. 1 (2018): 21–28. 29241748 10.1016/j.clindermatol.2017.09.005

[48] Z. J. Tang , J. R. Yang , C. L. Yu , M. H. Dong , R. Wang , and C. X. Li , “A Bibliometric Analysis of Global Research Trends in Psoriasis and Metabolic Syndrome,” Clinical, Cosmetic and Investigational Dermatology 17 (2024): 365–382. 38352064 10.2147/CCID.S446966 PMC10863501

[49] A. B. Kelesoglu Dincer and S. Sezer , “Systemic Immune Inflammation Index as a Reliable Disease Activity Marker in Psoriatic Arthritis,” Journal of the College of Physicians and Surgeons Pakistan 32, no. 6 (2022): 773–778. 35686411 10.29271/jcpsp.2022.06.773

[50] A. W. Armstrong , A. M. Soliman , K. A. Betts , et al., “Comparative Efficacy and Relative Ranking of Biologics and Oral Therapies for Moderate‐to‐Severe Plaque Psoriasis: A Network Meta‐analysis,” Dermatology and Therapy 11, no. 3 (2021): 885–905. 33788177 10.1007/s13555-021-00511-1 PMC8163943

[51] F. Bellinato , P. Gisondi , and G. Girolomoni , “Latest Advances for the Treatment of Chronic Plaque Psoriasis with Biologics and Oral Small Molecules,” Biologics: targets & therapy 15 (2021): 247–253. 34239295 10.2147/BTT.S290309 PMC8258237

[52] M. Ivanic , “Update on Biologics for Psoriasis in Clinical Practice,” Cutis. 108, no. 2S (2021): 15–18. 34662274 10.12788/cutis.0317

[53] L. Landeck , R. Sabat , K. Ghoreschi , et al., “Immunotherapy in psoriasis,” Immunotherapy. 13, no. 7 (2021): 605–619. 33820446 10.2217/imt-2020-0292

[54] K. A. Papp , M. A. Weinberg , A. Morris , and K. Reich , “IL17A/F nanobody sonelokimab in patients with plaque psoriasis: a multicentre, randomised, placebo‐controlled, phase 2b study,” The Lancet 397, no. 10284 (2021): 1564–1575. 10.1016/S0140-6736(21)00440-2 33894834

[55] E. Sbidian , A. Chaimani , I. Garcia‐Doval , et al., “Systemic pharmacological treatments for chronic plaque psoriasis: a network meta‐analysis,” The Cochrane Database of Systematic Reviews 5, no. 5 (2022): 011535. 10.1002/14651858.CD011535.pub5 PMC9125768 35603936

