# Supplementary material for: Association Between Systemic Immune‐Inflammation Index and Psoriasis, Psoriasis Comorbidities, and All‐Cause Mortality: A Study Based on NHANES
Source: Immun Inflamm Dis. 2024 Oct 28;12(10):e70050. doi: 10.1002/iid3.70050 (PMC11515906; doi:10.1002/iid3.70050)
Supplement: Supplementary file 1 — Supporting information. [file IID3-12-e70050-s001.docx]

**Table S1** Baseline characteristics of all psoriatic participants based on the severity of psoriasis

| **Variable** |  | **Severity** | | ***P***-value |
| --- | --- | --- | --- | --- |
|  | Total(N=429) | Mild(N=349) | Moderate to severe(N=80) |  |
| SII | 550.40(382.50,764.08) | 549.00(373.03,739.38) | 605.22(404.57,794.38) | 0.19 |
| Age | 47.00(0.87) | 47.43(0.88) | 45.11(2.28) | 0.32 |
| Poverty income ratio | 3.15(0.09) | 3.26(0.10) | 2.68(0.19) | **0.01** |
| BMI | 30.11(0.41) | 29.93(0.48) | 30.86(1.27) | 0.52 |
| Neutrophils | 4.30(3.20,5.60) | 4.20(3.20,5.50) | 4.30(3.50,6.10) | 0.14 |
| Lymphocyte | 2.00(1.60,2.50) | 2.00(1.60,2.50) | 2.00(1.50,2.40) | 0.76 |
| Platelet | 248.00(211.00,299.00) | 247.00(210.00,300.00) | 251.00(229.00,295.00) | 0.49 |
| Sex |  |  |  | 0.89 |
| female | 229(53.78) | 189(53.56) | 40(54.78) |  |
| male | 200(46.22) | 160(46.44) | 40(45.22) |  |
| Race/ethnicity, % |  |  |  | 0.35 |
| Non-Hispanic White | 257(81.04) | 207(80.82) | 50(81.97) |  |
| Mexican American | 29(3.38) | 22(2.87) | 7(5.61) |  |
| Non-Hispanic Black | 60(5.87) | 49(5.81) | 11(6.13) |  |
| Other Race | 52(5.73) | 44(5.93) | 8(4.86) |  |
| Other Hispanic | 31(3.98) | 27(4.57) | 4(1.43) |  |
| Education, % |  |  |  | 0.52 |
| above high school | 267(67.93) | 220(69.45) | 47(61.29) |  |
| below high school | 73(12.14) | 59(11.06) | 14(16.85) |  |
| high school | 89(19.93) | 70(19.49) | 19(21.87) |  |
| Drinking status, % |  |  |  | 0.05 |
| heavy drinker | 34( 9.82) | 29(10.72) | 5( 5.92) |  |
| low-to-moderate drinker | 314(74.85) | 256(76.04) | 58(69.65) |  |
| nondrinker | 81(15.33) | 64(13.24) | 17(24.43) |  |
| Smoking status, % |  |  |  | 0.59 |
| current smoker | 86(19.20) | 69(18.40) | 17(22.66) |  |
| former smoker | 137(34.72) | 110(35.98) | 27(29.26) |  |
| never smoker | 206(46.08) | 170(45.62) | 36(48.09) |  |
| History of CVD, % |  |  |  | **0.001** |
| No | 372(90.82) | 298(89.28) | 74(97.53) |  |
| Yes | 57( 9.18) | 51(10.72) | 6( 2.47) |  |
| History of MetS, % |  |  |  | **0.02** |
| No | 248(61.48) | 207(64.71) | 41(47.42) |  |
| Yes | 181(38.52) | 142(35.29) | 39(52.58) |  |
| Physical activity, % |  |  |  | 0.36 |
| insufficiently active | 177(44.60) | 149(46.64) | 28(35.70) |  |
| active | 153(35.64) | 124(34.97) | 29(38.56) |  |
| inactive | 99(19.76) | 76(18.39) | 23(25.75) |  |

Abbreviations: SII: Systemic immune-inflammation index; BMI: Body Mass Index (kg/m2); CVD: Cardiovascular Disease; MetS: Metabolic Syndrome

Normally distributed continuous variables are described as means ± SEs, and continuous variables without a normal distribution are presented as medians [interquartile ranges]. Categorical variables are presented as numbers (percentages). N reflect the study sample.

**Table S2.** Correlation analysis between SII and BSA severity of psoriasis.

| **Analysis** | **Crude Model** | |  | **Model I** | |  | **Model II** | |
| --- | --- | --- | --- | --- | --- | --- | --- | --- |
|  | **OR (95%CI)** | ***P*** |  | **OR (95%CI)** | ***P*** |  | **OR (95%CI)** | ***P*** |
| **Correlation analysis between SII and BSA severity** | | | | | | | | |
| **Continuous variable (log2SII)** | 1.26(0.91,1.74) | 0.16 |  | 1.27(0.92,1.75) | 0.15 |  | 1.13(0.78, 1.65) | 0.50 |
| **Categorical variable** |  |  |  |  |  |  |  |  |
| **Q1** | Reference |  |  | Reference |  |  | Reference |  |
| **Q2** | 1.42(0.56,3.62) | 0.45 |  | 1.53(0.59,3.98) | 0.37 |  | 1.45(0.56, 3.75) | 0.43 |
| **Q3** | 1.14(0.44,2.95) | 0.79 |  | 1.13(0.43,2.93) | 0.80 |  | 0.89(0.31, 2.52) | 0.82 |
| **Q4** | 1.71(0.71,4.11) | 0.23 |  | 1.80(0.73,4.43) | 0.19 |  | 1.39(0.55, 3.51) | 0.47 |
| **P for trend** |  | 0.289 |  |  | 0.296 |  |  | 0.733 |

Crude model adjusted for nothing, Model I adjusted for age, gender, ethnicity; Model II adjusted for age, gender, ethnicity, education, poverty, body mass index, smoking status, alcohol drinking status, physical activity and self-reported history of CVD. Q1 Range, [92,372.692]; Q2 Range, (372.692,547.5]; Q3 Range, (547.5,755.273]; Q4 Range, (755.273,3131].

**Table S3.** Baseline characteristics of participants with psoriasis based on MetS.

|  |  | **MetS** | |  |
| --- | --- | --- | --- | --- |
| **Variable** | **Total** (N=600) | **No** (N=336) | **Yes** (N=264) | ***P*-value** |
| SII | 545.24(374.00,746.67) | 514.76(359.60,678.46) | 596.70(414.27,835.71) | **< 0.001** |
| Age | 47.64(0.75) | 43.91(0.89) | 53.34(1.06) | **< 0.001** |
| Poverty income ratio | 3.14(0.08) | 3.28(0.10) | 2.93(0.12) | **0.03** |
| BMI | 29.98(0.33) | 27.68(0.41) | 33.48(0.47) | **< 0.001** |
| Neutrophils | 4.30(3.20,5.50) | 4.00(3.00,5.20) | 4.70(3.60,5.70) | **< 0.001** |
| Lymphocyte | 2.00(1.50,2.50) | 1.90(1.60,2.50) | 2.00(1.50,2.50) | 0.61 |
| Platelet | 241.00(209.00,294.00) | 235.00(200.00,284.00) | 251.00(219.00,306.00) | 0.04 |
| Sex, % |  |  |  | 0.64 |
| Female | 315(51.86) | 176(50.95) | 139(53.23) |  |
| Male | 285(48.14) | 160(49.05) | 125(46.77) |  |
| Race/ethnicity, % |  |  |  | 0.54 |
| Mexican American | 48( 3.70) | 22(3.41) | 26(4.15) |  |
| Other Hispanic | 46( 3.84) | 25(4.16) | 21(3.35) |  |
| Non-Hispanic White | 370(81.07) | 209(80.40) | 161(82.09) |  |
| Non-Hispanic Black | 75( 5.81) | 40(5.46) | 35(6.34) |  |
| Other race | 61( 5.58) | 40(6.58) | 21(4.06) |  |
| Education, % |  |  |  | **0.01** |
| Below high school | 120(13.94) | 56(11.17) | 64(18.19) |  |
| High school | 134(21.13) | 65(18.54) | 69(25.08) |  |
| Above high school | 346(64.93) | 215(70.29) | 131(56.73) |  |
| Smoking status, % |  |  |  | 0.09 |
| Nondrinker | 111(15.71) | 50(12.37) | 61(20.82) |  |
| Low-to-moderate drinker | 432(73.72) | 253(75.94) | 179(70.33) |  |
| Heavy drinker | 57(10.57) | 33(11.69) | 24( 8.85) |  |
| Smoking status, % |  |  |  | **0.01** |
| Never smoker | 264(43.32) | 165(48.28) | 99(35.74) |  |
| Former smoker | 196(35.48) | 89(29.89) | 107(44.04) |  |
| Current smoker | 140(21.19) | 82(21.83) | 58(20.21) |  |
| History of CVD, % |  |  |  | **< 0.001** |
| No | 512(89.91) | 310(94.81) | 202(82.42) |  |
| Yes | 88(10.09) | 26( 5.19) | 62(17.58) |  |
| Physical activity, % |  |  |  | **0.001** |
| Inactive | 155(21.24) | 69(15.82) | 86(29.53) |  |
| Insufficiently active | 231(42.40) | 127(42.92) | 104(41.60) |  |
| Active | 214(36.36) | 140(41.26) | 74(28.87) |  |
| History of arthritis % |  |  |  | **< 0.001** |
| No | 353(62.03) | 234(69.96) | 119(49.91) |  |
| Yes | 247(37.97) | 102(30.04) | 145(50.09) |  |

Abbreviations: SII: Systemic immune-inflammation index; BMI: Body Mass Index (kg/m2); CVD: Cardiovascular Disease; MetS: Metabolic Syndrome

Normally distributed continuous variables are described as means ± SEs, and continuous variables without a normal distribution are presented as medians [interquartile ranges]. Categorical variables are presented as numbers (percentages). N reflect the study sample.

**Table S4.** Correlation analysis between SII and CVD or arthritis among psoriasis patients.

| **Analysis** | **Crude Model** | |  | **Model I** | |  | **Model II** | |
| --- | --- | --- | --- | --- | --- | --- | --- | --- |
|  | **OR (95%CI)** | ***P*** |  | **OR (95%CI)** | ***P*** |  | **OR (95%CI)** | ***P*** |
| **Correlation analysis between SII and CVD** | | | | | | | | |
| **Continuous variable (log2SII)** | 0.79(0.44,1.40) | 0.41 |  | 0.82(0.49, 1.37) | 0.45 |  | 0.70(0.42, 1.16) | 0.16 |
| **Categorical variable** |  |  |  |  |  |  |  |  |
| **Q1** | Reference |  |  | Reference |  |  | Reference |  |
| **Q2** | 0.49(0.22,1.05) | 0.07 |  | 0.42(0.18, 0.99) | 0.05 |  | 0.36(0.14, 0.92) | 0.03 |
| **Q3** | 0.53(0.23,1.21) | 0.13 |  | 0.57(0.23, 1.42) | 0.22 |  | 0.57(0.24, 1.35) | 0.20 |
| **Q4** | 0.58(0.24,1.37) | 0.21 |  | 0.54(0.22, 1.34) | 0.18 |  | 0.33(0.13, 0.86) | 0.02 |
| **P for trend** |  | 0.251 |  |  | 0.29 |  |  | 0.059 |
| **Correlation analysis between SII and arthritis** | | | | | | | | |
| **Continuous variable (log2SII)** |  |  |  |  |  |  |  |  |
| **Categorical variable** |  |  |  |  |  |  |  |  |
| **Q1** | Reference |  |  | Reference |  |  | Reference |  |
| **Q2** | 1.48(0.80,2.75) | 0.20 |  | 1.44(0.72,2.91) | 0.30 |  | 1.37(0.64, 2.90) | 0.41 |
| **Q3** | 1.28(0.77,2.14) | 0.34 |  | 1.39(0.77,2.51) | 0.27 |  | 1.31(0.68, 2.52) | 0.42 |
| **Q4** | 1.52(0.79,2.96) | 0.21 |  | 1.46(0.74,2.87) | 0.27 |  | 1.33(0.65, 2.72) | 0.43 |
| **P for trend** |  | 0.315 |  |  | 0.315 |  |  | 0.486 |

Crude model adjusted for nothing, Model I adjusted for age, gender, ethnicity; Model II adjusted for age, gender, ethnicity, education, poverty, body mass index, smoking status, alcohol drinking status, physical activity and self-reported history of CVD apart from stratification factor itself. Q1 Range, [92,366.667]; Q2 Range, (366.667,525.709]; Q3 Range, (525.709,746.069]; Q4 Range, (746.069,3131].


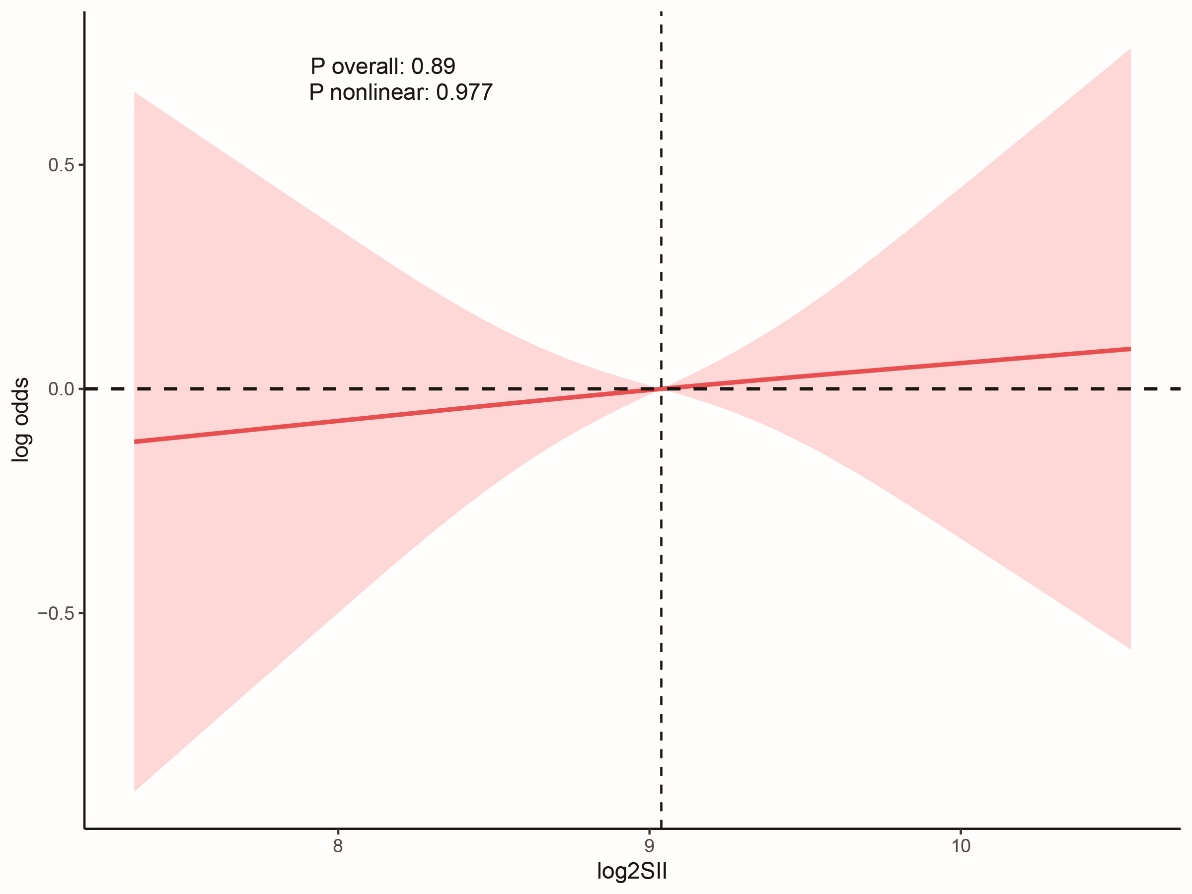


**FigureS1** Restricted cubic spline analyses the association of between SII and arthritis in psoriasis patients. Adjusted for age, gender, ethnicity, education, poverty, body mass index, smoking status, alcohol drinking status, physical activity and self-reported history of CVD. Abbreviations: SII, Systemic immune-inflammation index. CVD, cardiovascular disease.


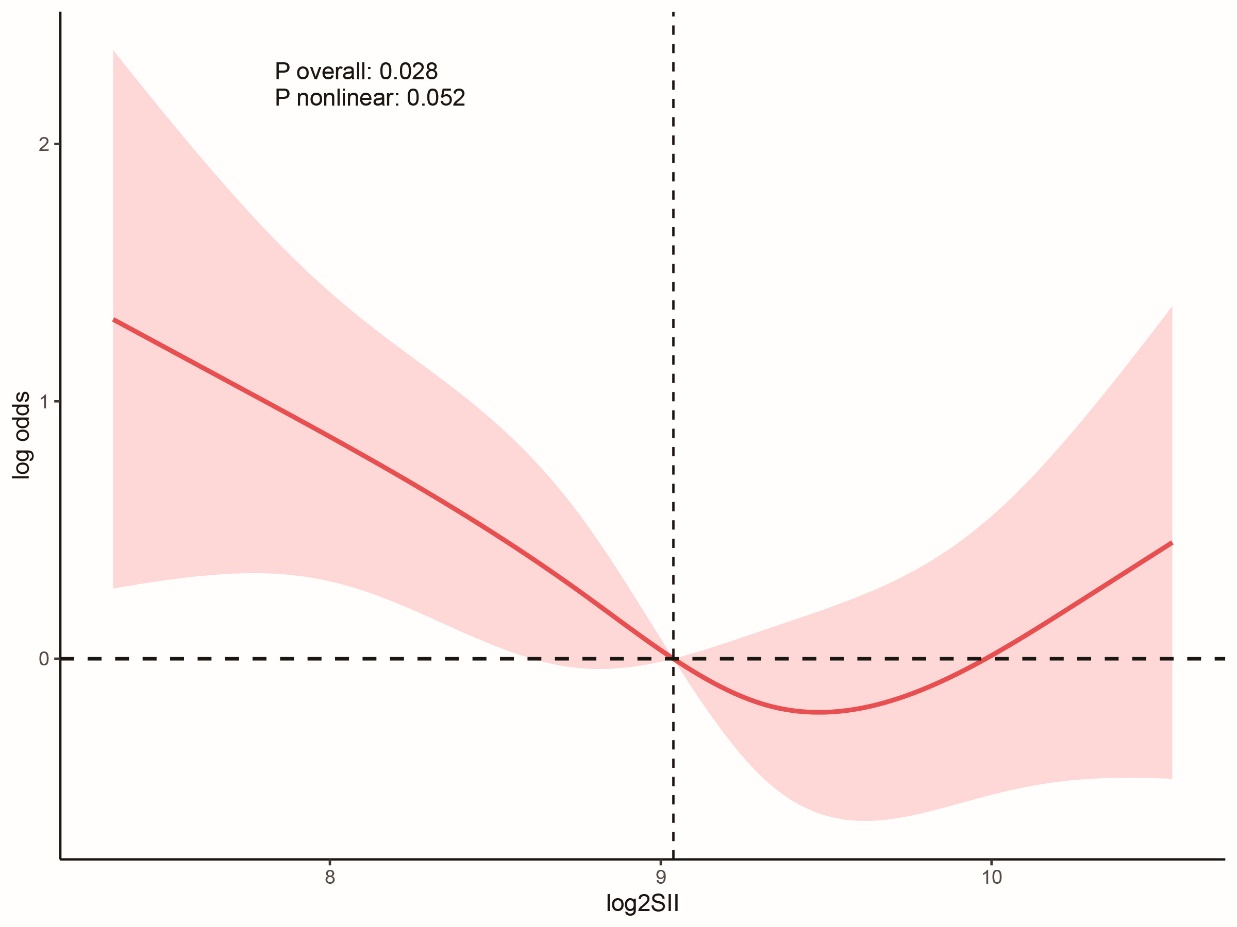


**FigureS2** Restricted cubic spline analyses the association of between SII and CVD in psoriasis patients. Adjusted for age, gender, ethnicity, education, poverty, body mass index, smoking status, alcohol drinking status, physical activity. Abbreviations: SII, Systemic immune-inflammation index. CVD, cardiovascular disease.
